# Changes in Neutrophil to Lymphocyte Ratio, Lymphocyte to Monocyte Ratio, and Platelet to Lymphocyte Ratio During Palliative Radiotherapy May Predict Efficacy of Immune Checkpoint Inhibitor as Re-Challenge Treatment in Advanced Gastric Cancer: A Case Report

**DOI:** 10.3389/fonc.2022.873213

**Published:** 2022-05-19

**Authors:** Jianxin Chen, Xilin Wu, Shijian Zhu, Junhui Wang

**Affiliations:** The Quzhou Affiliated Hospital of Wenzhou Medical University, Quzhou People’s Hospital, Quzhou, China

**Keywords:** gastric cancer, re-challenge, neutrophil to lymphocyte ratio, programmed death-1 inhibitor, lymphocyte to monocyte ratio

## Abstract

**Introduction:**

Immunotherapy with programmed death-1 (PD-1) inhibitors has emerged as frontline option in patients with advanced or metastatic gastric cancer. However, two-thirds of patients who received PD-1 inhibitors treatment still had disease progression in 1 year. Subsequent treatment strategies as salvage options always lead to limited efficacy.

**Case Description:**

Herein, we presented a case of recurrent metastatic gastric adenocarcinoma that had progressed on first-line treatment with nivolumab, in which systematic inflammation parameters with neutrophil to lymphocyte ratio (NLR), lymphocyte to monocyte ratio (LMR), and platelet to lymphocyte ratio (PLR) were significantly changed by palliative radiotherapy on metastatic lymph nodes. The patient achieved persistent response to the re-challenge of immune checkpoint inhibitor, which resulted in survival time reaching 52 months, and is still in extension.

**Conclusions:**

We supposed that the palliative radiotherapy may lead to the correction of NLR, LMR, and PLR and finally contribute to the efficacy of the re-challenge treatment by PD-1 inhibitor.

## Introduction

Gastric cancer constitutes a global health problem, with more than one million new cases and 768,000 deaths in 2020, making it the fifth most frequently diagnosed cancer and the third leading cause of cancer-related deaths all over the world (1, 2). Patients with gastric cancer always had a poor prognosis, since gastric cancer is generally diagnosed at an advanced or metastatic stage, with limited opportunity to receive radical operation. The median survival time for patients diagnosed with advanced or metastatic disease is <12 months (3, 4). It should be an urgent need to prolong the survival time in such patients.

Nivolumab, a fully human IgG4 monoclonal antibody inhibitor of programmed death-1, has emerged as the first one to show superior survival time, with a median overall survival time exceeding 1 year in the first-line setting for patients with non-HER2-positive gastric, gastroesophageal junction, or esophageal adenocarcinoma (5). However, 64% of patients who received nivolumab plus chemotherapy as first-line regimen still had disease progression in 1 year (5). Subsequent monotherapy with pembrolizumab, another immune checkpoint inhibitor, has failed to improve survival time compared with paclitaxel for advanced gastric or gastroesophageal junction cancer with PD-L1 CPS of 1 or higher (6). The current efficacy of further line treatment strategies could not satisfy clinical demand.

In most recent years, it is reported that the re-challenge of PD-1/PD-L1 inhibitors as further line treatment might still be effective in selective patients (7). However, few studies were conducted to investigate the specific profiting population, with the mechanism of re-challenge remaining to be explored. A recent retrospective pooled analysis demonstrated that high pretreatment neutrophil to lymphocyte ratio (NLR) was significantly associated with poorer progression-free survival time (PFS, HR = 1.44, 95% CI 1.26–1.65; *p* < 0.001) and overall survival time (OS, HR = 2.86, 95% CI 2.11–3.87; *p* < 0.001) compared with those with low pretreatment NLR in patients with non-small cell lung cancer (8). In addition, similar outcomes were also observed in metastatic melanoma and renal cell carcinoma (9, 10). Even so, a majority of publications focused on the relationship between pretreated level of systematic inflammation markers including NLR, lymphocyte to monocyte ratio (LMR), and platelet to lymphocyte ratio (PLR), and clinical outcomes. Few studies have been conducted to investigate the strategies to correct the level of NLR, LMR, and PLR, aiming to improve the clinical outcomes. Herein, we presented a case of recurrent metastatic gastric adenocarcinoma that had progressed on first-line treatment with nivolumab, in which systematic inflammation parameters with NLR, LMR, and PLR were changed by palliative radiotherapy on metastatic lymph nodes. The patient achieved persistent response to the re-challenge of immune checkpoint inhibitor, which resulted in survival time reaching 52 months, and is still in extension. We supposed that the palliative radiation may lead to the correction of NLR, LMR, and PLR and finally contributed to the persistent efficacy of the re-challenge treatment by PD-1 inhibitor.

## Case Description

**Figure 1:**
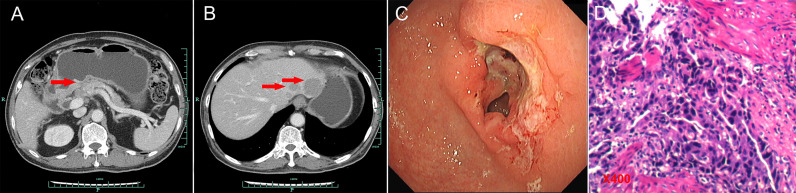
Abdominal CT scans and gastroscopy findings at baseline. **(A)** Abdominal CT scans suggested thickened gastric wall in the antrum (red arrow for tumor). **(B)** Abdominal CT scans showed liver metastasis (red arrows for tumors). **(C)** Gastroscopy examinations revealed gastric ulcer. **(D)** Histological finding with hematoxylin and eosin–stained biopsy specimen from gastroscopy (400×).

**Figure 2:**
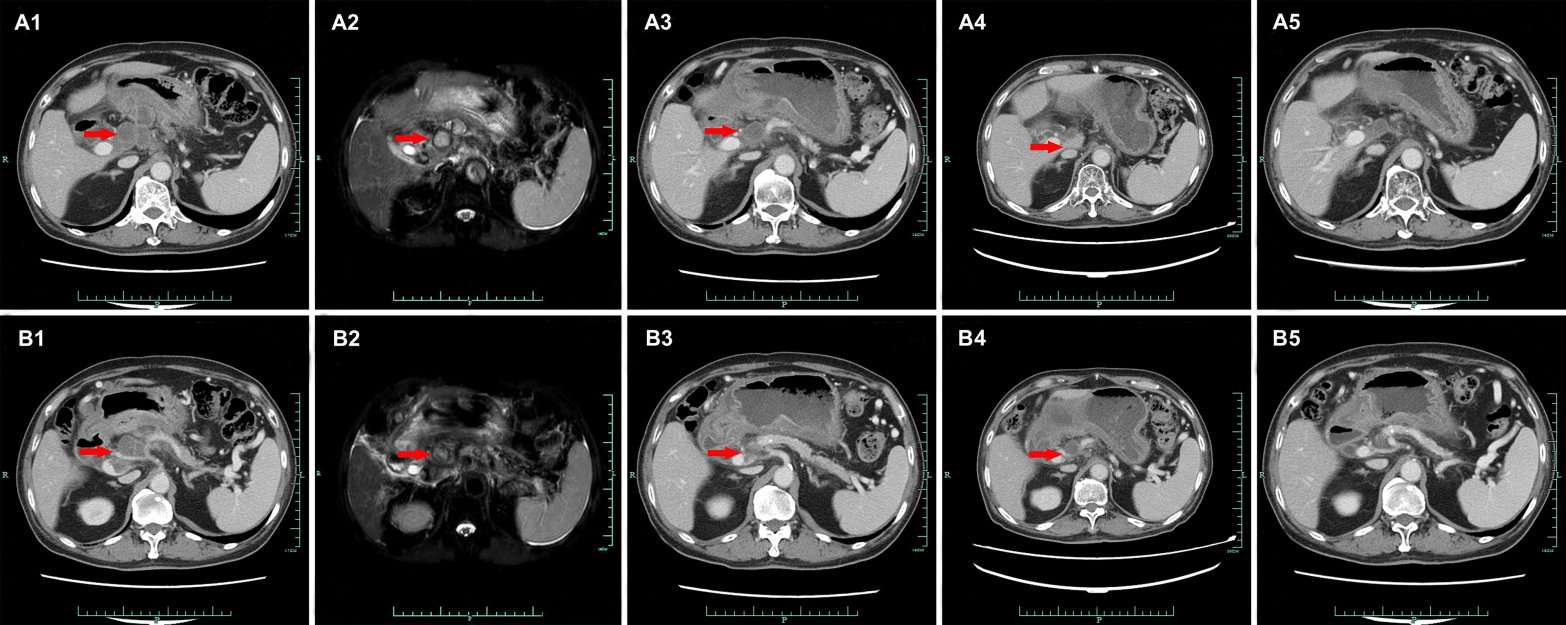
Abdominal CT presentations after recurrence (from July 11, 2020 to August 4, 2021, red arrows for tumors). (**A1/B1**) July 11, 2020. (**A2/B2**) October 22, 2020. (**A3/B3**) January 25, 2021. (**A4/B4**) April 6, 2021. (**A5/B5**) August 4, 2021.

**Figure 3:**
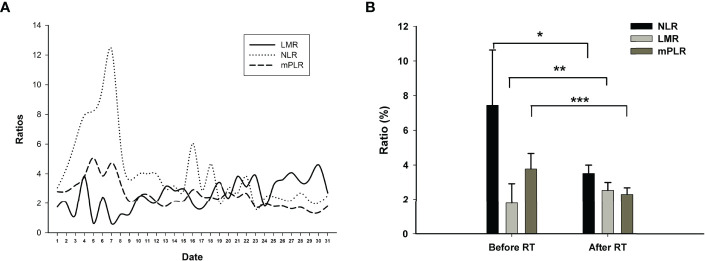
**(A)** The variations in ratios for LMR (lymphocyte to macrophages ratio), NLR (neutrophils to lymphocyte ratio), and mPLR (modified platelet to lymphocyte ratio, platelet to lymphocyte ratio/50) from July 3, 2020 (No. 1 at x-axis) to August 31, 2021 (No. 31 at x-axis). **(B)** The means of LMR, NLR, and mPLR before radiotherapy (2 months before radiotherapy) and after radiotherapy (2 months from the beginning of radiotherapy). *p = 0.016, **p = 0.037, ***p = 0.002.

A 74-year-old Chinese man was admitted to our hospital on September 11, 2017 with repeated abdominal distension for 3 months. The patient denied smoking, alcohol, or any other medical or family history. Abdominal CT showed thickened gastric wall in the antrum and surrounded by enlarged lymph nodes (**Figure 1A**), along with multiple lesions in liver, suggesting metastasis (**Figure 1B**). Subsequently, the patient received gastroscopy examination, the results of which revealed a large ulcer (3.5 cm × 3.5 cm), with dirty surface and scattered bleeding spots (**Figure 1C**). Based on the biopsy findings by gastroscopy, the patient was diagnosed as having gastric adenocarcinoma, with metastasis on interstitial lymph nodes of the liver and stomach, and liver (**Figure 1D**). Immunohistochemistry outcomes were presented as CDX2 (positive), CK18 (positive), CK19 (positive), CEA (negative), CK20 (negative), and Her-2 (negative). Additionally, results of next-generation sequencing (NGS) using tissues revealed microsatellite stability (MSS), programmed cell death ligand-1 (PD-L1) negative, and low tumor mutation burden (TMB). His palliative therapy was started with regimen XELOX (oxaliplatin of 130 mg/m^2^ on day 1 and oral capecitabine of 1,000 mg/m^2^ twice a day, from day 1 to 14, every 21 days) combined with immune checkpoint inhibitor nivolumab on September 15, 2017. After three cycles’ exposure of the treatment strategy, the efficacy was evaluated as partial response (PR), with complete response (CR) for lesions on liver according to the criteria of Response Evaluation Criteria in Solid Tumors (RECIST) version 1.1. He finally completed six cycles of first-line treatment, with manageable toxicities, and subsequently received maintenance therapy with mono-nivolumab. However, after 2 months of maintenance, significantly elevated glutamic-pyruvic transaminase (ALT = 637 U/ml) was detected, which was considered as immunotherapy-related hepatitis. Based on that, maintenance therapy was terminated, but follow-up continued. On July 11, 2020, his regular abdomen CT showed re-enlargement and fused interstitial lymph nodes of the liver and stomach (**Figure 2A1**), suggesting progressive disease (PD). Metastatic lymph nodes invaded the portal and splenic veins (**Figure 2B1**). The symptom of abdominal distension appeared again, leading to performance status (PS) being degraded to 3. The patient refused any cytotoxic medication treatment but agreed to receive palliative medical care. After discussion by the multidisciplinary team including medical oncologist, surgical oncologist, radiation oncologist, and medical imaging doctors, intensity modulated radiotherapy (IMRT) on enlarged and fused interstitial lymph nodes of the liver and stomach was finally adopted as symptomatic care. Radiation schedule was set as 95% planning-gross target volume (P-GTV), with a total of 50 Gy for 25 times. The palliative radiation was started on August 11, 2020 and ended on September 19, 2020. Abdominal distension was relieved. Efficacy assessment for radiation was conducted by abdomen CT scan in October 2020 (**Figures 2A2,B2**), which suggested a stable disease (SD, by 16% regression for target lymph nodes). During the radiotherapy, we surprisingly detected a dynamic change in elevated level of LMR, accompanied with lowered level of NLR and PLR (**Figure 3A**), which suggested a potential response to systematic therapy with immune checkpoint inhibitor (11–14). In addition, we also observed a significant variation in the means of LMR, NLR, and mPLR (PLR/50) before radiotherapy (2 months before radiotherapy) and after radiotherapy (2 months from the beginning of radiotherapy), the results of which are presented in **Figure 3B**. Based on this, re-challenge treatment of PD-1 inhibitor was considered as consolidation therapy. However, due to the elevated ALT observed during the treatment of nivolumab, monotherapy of sintilimab, another PD-1 inhibitor, was prescribed as maintenance treatment from November 20, 2020. After 2 months treatment of sintilimab, repeat abdomen CT scan revealed a partial response (PR, by 64% regression for target lymph nodes; **Figures 2A3, B3**). The latest CT scan was performed on August 4, 2021, the results of which still suggested a continuous PR, with complete regression on portal vein and splenic vein (by 67% regression for target lymph nodes; **Figures 2A5, B5**). There was no treatment-related adverse event observed during the administration of sintilimab. The patient still received the regimen regularly, with overall survival time of 52 months. The variation in tumor size for target lymph nodes is presented in **Table 1**. It should be noticed that the main gastric tumor was presented as thickened gastric wall with ulceration. There was no clear boundary for the thickened gastric wall with ulceration, which caused difficulty in measurement. That was the reason why the enlarged and fused interstitial lymph nodes of the liver and stomach, rather than the main gastric tumor, were selected as target lesion. In addition, the timeline with relevant data during the treatment is presented in **Table 2**.

**Table 1:** Variations of enlarged lymph nodes size after recurrence according to RECIST version 1.1.

Date	Exposure	Duration	Enlarged lymph nodes of A area (LD×SD, mm)	Enlarged lymph nodes of B area (LD×SD, mm)
2020.07	Radiation		54×39	43×33
2020.10	Radiation		39×29	42×25
2021.01	Sintilimab	2 month	36×17	26×21
2021.04	Sintilimab	5 months	27×17	17×16
2021.08	Sintilimab	9 months	18×15	14×13

LD, longest diameter; SD, shortest diameter. A area, enlarged lymph nodes of A1–A5 in **Figure 2**. B area, enlarged lymph nodes of B1–B5 in **Figure 2**.

**Table 2:** Timeline with relevant data during the treatment.

Timeline	Intervention	Best response	Adverse events
September 2017 to February 2018	Nivolumab, Xelox	PR	Neutropenia grade 2
March 2018 to May 2018	Nivolumab	PR	Elevated ALT grade 3
June 2018 to July 2020	Follow-up	NA	NA
August 2020 to September 2020	Radiotherapy	SD	NO
November 2020 to present	Sintilimab	PR	NO

Xelox, Oxaliplatin, Capecitabine; PR, partial response; NA, not applicable; SD, stable disease; NO, not observed; ALT, glutamic-pyruvic transaminase.

## Discussion

We herein presented a case of recurrent metastatic gastric adenocarcinoma, in which systematic inflammation parameters with NLR, LMR, and PLR have been changed by palliative radiotherapy and has achieved persistent response to the re-challenge of immune checkpoint inhibitor, with survival time reaching 52 months, and is still in extension.

In recent years, systematic inflammation markers including NLR, LMR, and PLR have been reported as indicators of systematic response for cancer treatment strategies. High NLR and PLR and low LMR are supposed as markers of unspecific immune system activation, correlating significant poor clinical outcomes in various cancers including advanced gastric cancer patients (15), small cell lung cancer (16), colorectal cancers (17, 18), pancreatic cancer (19), and non-small cell lung cancers (8). However, in previous literatures, only internal factors including NLR, LMR, and PLR, which were evaluated before systematic treatment, had been adequately investigated. For patients with possible negative prognostic parameters (higher NLR, higher PLR, and lower LMR, without a standard range), few studies have been conducted to explore strategies to correct the unspecific immune system activation, such as lowering the level of NLR and PLR and improving the level of LMR. In recent decades, published literatures suggested a significant association between tumor response and treatment-related neutropenia and thrombocytopenia (20–22). In a pooled analysis, researchers performed a *post-hoc* analysis pooling data prospectively collected in six randomized phase III trials in non-small cell lung cancer. In multivariable overall survival (OS) analysis, chemotherapy-induced neutropenia (CIN) was significantly predictive of prognosis (hazard ratio of death, 0.71; 95%CI: 0.53–0.95) (22), which might be caused by the decline in NLR, as lymphocytes were little influenced by cytotoxic agents (23–25). In addition, NLR was also reported as a meaningful predictor of outcome in patients with extensive small-cell lung cancer who received radiation treatment (26). It was reported that radiotherapy also reduced the level of NLR, which may contribute to a longer survival time, even in patients with advanced or metastatic disease (27). Although the above clinical evidence reported a meaningful predictive value of NLR/LMR/PLR in practice, few basic experiments were conducted to investigate the intrinsic mechanism for the phenomenon. According to the varied data of NLR in the present case, we detected that the decrease in neutrophil might be the dominant reason for the decrease in NLR. We supposed that the count of neutrophil, rather than lymphocyte, may be easier influenced by anti-tumor treatment including chemotherapy or radiotherapy. We speculated that the ratio of circulating neutrophils to lymphocytes was supposed to correlate with the interaction between inflammation and immunity, especially the potential mechanism in tumor microenvironment. The potential mechanism of the interesting clinical phenomenon has been discussed with our basic experimental researchers. We hope that there would be something to be discovered by the cooperation of basic and clinical findings in the future. Even so, parameters correction (NLR/PLR/LMR) by systematic or local treatment is supposed to be beneficial for the improvement of clinical outcomes, which is also suggested in our study. In the present case, we scheduled palliative radiation on the metastatic fused interstitial lymph nodes of the liver and stomach nodes. As a result, we amazingly found the correction of immune systematic parameters including the reduction in NLR and PLR and the improvement of LMR (**Figure 3A**). Based on this, re-challenge of immunotherapy was administrated, which may have finally led to the persistent response in the present case.

Most recently, there were several retrospective studies that reported the efficacy and safety of the re-challenge of PD-1/PD-L1 inhibitors (7, 28), the results of which indicate that the effectiveness of treatment with a PD-1 inhibitor is maintained during retreatment and that this may be a valid therapeutic option for routine clinical practice in selected patients. Although the mechanism of PD-1 inhibitors re-challenge still remains uncovered, authors described that the patients who received a second course of PD-1 inhibitor after an intervening course of standard chemotherapy presented a better survival time compared to a drug holiday following nivolumab discontinuation (median OS of 18.1 versus 14.8 months) (7). However, it was not mentioned whether or not the variation in immune systematic parameters (NLR/PLR/LMR) contributes to the difference between the groups (7). Even so, with the superior efficacy presented in the present case, we still supposed that intervening treatment with radiotherapy may lead to the correction of immune systematic parameters, which might finally contribute to the response of cancer and the extension of survival time.

The leading limitation of the present case report comes from the nature of a case report. Although we supposed that the improvement of systematic inflammation parameters with NLR, PLR, and LMR may contribute to the re-challenge of immune checkpoint inhibitor in advanced gastric cancer, the conclusion still needs further identification in prospective clinical practice and basic experiments.

Briefly, we presented a case of recurrent metastatic gastric adenocarcinoma, whose systematic inflammation parameters with NLR, LMR, and PLR were changed by palliative radiation and has achieved persistent response to the re-challenge of immune checkpoint inhibitor, with survival time reaching 52 months, and is still in extension.

## Data Availability Statement

The raw data supporting the conclusions of this article will be made available by the authors, without undue reservation.

## Ethics Statement

The studies involving human participants were reviewed and approved by the Ethical Committee of People’s Hospital of Quzhou. The patients/participants provided their written informed consent to participate in this study.

## Author Contributions

JC contributed to conception and design of the study. XW organized the database. SZ performed the statistical analysis. JC wrote the first draft of the manuscript. SZ and JW wrote sections of the manuscript. All authors contributed to the article and approved the submitted version.

## Conflict of Interest

The authors declare that the research was conducted in the absence of any commercial or financial relationships that could be construed as a potential conflict of interest.

## Publisher’s Note

All claims expressed in this article are solely those of the authors and do not necessarily represent those of their affiliated organizations, or those of the publisher, the editors and the reviewers. Any product that may be evaluated in this article, or claim that may be made by its manufacturer, is not guaranteed or endorsed by the publisher.

## References

[1] Siegel RL Miller KD Jemal A. Cancer Statistics, 2020. CA Cancer J Clin (2020) 70:7–30. doi: 10.3322/caac.21590 31912902

[2] World Health Organization. International Agency for Research on Cancer. In: GLOBOCAN 2020: Stomach Cancer Fact Sheet (2020). Available at: https://gco.iarc.fr/today/data/factsheets/cancers/7-Stomach-fact-sheet.pdf.

[3] Bang YJ Van Cutsem E Feyereislova A Chung HC Shen L Sawaki A. Trastuzumab in Combination With Chemotherapy Versus Chemotherapy Alone for Treatment of HER2-Positive Advanced Gastric or Gastro-Oesophageal Junction Cancer (ToGA): A Phase 3, Open-Label, Randomised Controlled Trial. Lancet (2010) 376:687–97. doi: 10.1016/S0140-6736(10)61121-X 20728210

[4] Song J Lee HJ Cho GS Han SU Kim MC Ryu SW. Recurrence Following Laparoscopy-Assisted Gastrectomy for Gastric Cancer: A Multicenter Retrospective Analysis of 1,417 Patients. Ann Surg Oncol (2010) 17:1777–86. doi: 10.1245/s10434-010-0932-4 20151217

[5] Janjigian YY Shitara K Moehler M Garrido M Salman P Shen L. First-Line Nivolumab Plus Chemotherapy Versus Chemotherapy Alone for Advanced Gastric, Gastro-Oesophageal Junction, and Oesophageal Adenocarcinoma (CheckMate 649): A Randomised, Open-Label, Phase 3 Trial. Lancet (2021) 398:27–40. doi: 10.1016/S0140-6736(21)00797-2 34102137 PMC8436782

[6] Shitara K Ozguroglu M Bang YJ Di Bartolomeo M Mandala M Ryu MH. Pembrolizumab Versus Paclitaxel for Previously Treated, Advanced Gastric or Gastro-Oesophageal Junction Cancer (KEYNOTE-061): A Randomised, Open-Label, Controlled, Phase 3 Trial. Lancet (2018) 392:123–33. doi: 10.1016/S0140-6736(18)31257-1 29880231

[7] Giaj Levra M Cotte FE Corre R Calvet C Gaudin AF Penrod JR. Immunotherapy Rechallenge After Nivolumab Treatment in Advanced non-Small Cell Lung Cancer in the Real-World Setting: A National Data Base Analysis. Lung Cancer (2020) 140:99–106. doi: 10.1016/j.lungcan.2019.12.017 31911324

[8] Li Y Zhang Z Hu Y Yan X Song Q Wang G. Pretreatment Neutrophil-To-Lymphocyte Ratio (NLR) May Predict the Outcomes of Advanced Non-Small-Cell Lung Cancer (NSCLC) Patients Treated With Immune Checkpoint Inhibitors (ICIs). Front Oncol (2020) 10:654. doi: 10.3389/fonc.2020.00654 32656072 PMC7324627

[9] Ferrucci PF Gandini S Battaglia A Alfieri S Di Giacomo AM Giannarelli D. Baseline Neutrophil-to-Lymphocyte Ratio is Associated With Outcome of Ipilimumab-Treated Metastatic Melanoma Patients. Br J Cancer (2015) 112:1904–10. doi: 10.1038/bjc.2015.180 PMC4580390 26010413

[10] Kuzman JA Stenehjem DD Merriman J Agarwal AM Patel SB Hahn AW. Neutrophil-Lymphocyte Ratio as a Predictive Biomarker for Response to High Dose Interleukin-2 in Patients With Renal Cell Carcinoma. BMC Urol (2017) 17:1. doi: 10.1186/s12894-016-0192-0 28056941 PMC5217571

[11] Yang T Hao L Yang X Luo C Wang G Lin Cai C. Prognostic Value of Derived Neutrophil-to-Lymphocyte Ratio (dNLR) in Patients With Non-Small Cell Lung Cancer Receiving Immune Checkpoint Inhibitors: A Meta-Analysis. BMJ Open (2021) 11:e049123. doi: 10.1136/bmjopen-2021-049123 PMC8413941 34475167

[12] Vinal D Gutierrez-Sainz L Martinez D Garcia-Cuesta JA Pedregosa J Villamayor J. Prognostic Value of Neutrophil-to-Lymphocyte Ratio in Advanced Cancer Patients Receiving Immunotherapy. Clin Trans Oncol Off Publ Fed Spanish Oncol Soc Natl Cancer Inst Mexico (2021) 23:1185–92. doi: 10.1007/s12094-020-02509-1 33226553

[13] Shang J Han X Zha H Tao H Li X Yuan F. Systemic Immune-Inflammation Index and Changes of Neutrophil-Lymphocyte Ratio as Prognostic Biomarkers for Patients With Pancreatic Cancer Treated With Immune Checkpoint Blockade. Front Oncol (2021) 11:585271. doi: 10.3389/fonc.2021.585271 33718140 PMC7943876

[14] Fujimoto A Toyokawa G Koutake Y Kimura S Kawamata Y Fukuishi K. Association Between Pretreatment Neutrophil-to-Lymphocyte Ratio and Immune-Related Adverse Events Due to Immune Checkpoint Inhibitors in Patients With Non-Small Cell Lung Cancer. Thorac Cancer (2021) 12:2198–204. doi: 10.1111/1759-7714.14063 PMC8327687 34173724

[15] Konopka K Micek A Ochenduszko S Streb J Potocki P Kwinta L. Combined Neutrophil-To-Lymphocyte and Platelet-Volume-To-Platelet Ratio (NLR and PVPR Score) Represents a Novel Prognostic Factor in Advanced Gastric Cancer Patients. J Clin Med (2021) 10. doi: 10.3390/jcm10173902 PMC8432226 34501353

[16] Chen C Yang H Cai D Xiang L Fang W Wang R. Preoperative Peripheral Blood Neutrophil-to-Lymphocyte Ratios (NLR) and Platelet-to-Lymphocyte Ratio (PLR) Related Nomograms Predict the Survival of Patients With Limited-Stage Small-Cell Lung Cancer. Transl Lung Cancer Res (2021) 10:866–77. doi: 10.21037/tlcr-20-997 PMC7947425 33718028

[17] Ergen SA Barlas C Yildirim C Oksuz DC. Prognostic Role of Peripheral Neutrophil-Lymphocyte Ratio (NLR) and Platelet-Lymphocyte Ratio (PLR) in Patients With Rectal Cancer Undergoing Neoadjuvant Chemoradiotherapy. J Gastrointest Cancer (2021) 53:151–60. doi: 10.1007/s12029-020-00578-7 33392960

[18] Naszai M Kurjan A Maughan TS. The Prognostic Utility of Pre-Treatment Neutrophil-to-Lymphocyte-Ratio (NLR) in Colorectal Cancer: A Systematic Review and Meta-Analysis. Cancer Med (2021) 10:5983–97. doi: 10.1002/cam4.4143 PMC8419761 34308567

[19] Zhou L Wang J Zhang XX Lyu SC Pan LC Du GS. Prognostic Value of Preoperative NLR and Vascular Reconstructive Technology in Patients With Pancreatic Cancer of Portal System Invasion: A Real World Study. Front Oncol (2021) 11:682928. doi: 10.3389/fonc.2021.682928 34604028 PMC8484969

[20] Chabot GG Abigerges D Catimel G Culine S de Forni M Extra JM. Population Pharmacokinetics and Pharmacodynamics of Irinotecan (CPT-11) and Active Metabolite SN-38 During Phase I Trials. Ann Oncol (1995) 6:141–51. doi: 10.1093/oxfordjournals.annonc.a059109 7786822

[21] Osumi H Shinozaki E Ooki A Wakatsuki T Kamiimabeppu D Sato T. Early Hypertension and Neutropenia are Predictors of Treatment Efficacy in Metastatic Colorectal Cancer Patients Administered FOLFIRI and Vascular Endothelial Growth Factor Inhibitors as Second-Line Chemotherapy. Cancer Med (2021) 10:615–25. doi: 10.1002/cam4.3638 PMC7877370 33347731

[22] Gargiulo P Arenare L Gridelli C Morabito A Ciardiello F Gebbia V. Chemotherapy-Induced Neutropenia and Treatment Efficacy in Advanced Non-Small-Cell Lung Cancer: A Pooled Analysis of 6 Randomized Trials. BMC Cancer (2021) 21:549. doi: 10.1186/s12885-021-08323-4 33985435 PMC8120920

[23] Cho MY Joh YG Kim NR Jung SI Bae JW Kim YC. T-Lymphocyte Subsets in Patients With AJCC Stage III Gastric Cancer During Postoperative Adjuvant Chemotherapy. American Joint Committee on Cancer. Scand J Surg (2002) 91:172–7. doi: 10.1177/145749690209100207 12164518

[24] Wang Y Qu H Xu B Wu J Lu K Liu C. Expression of FOXA1 Is Associated With the Tumor-Infiltrating M2 Macrophage, Cytotoxic T Lymphocyte, and Effect of Chemotherapy in Bladder Cancer. Urol Int (2021), 1–6. doi: 10.1159/000519129 PMC9909707 34706362

[25] Ishibashi Y Tsujimoto H Hiraki S Kouzu K Tsuchiya S Itazaki Y. Predictive Value of Immuno-Inflammatory and Nutritional Measures Modulated by Neoadjuvant Chemotherapy on the Response of Neoadjuvant Chemotherapy and Long-Term Outcomes in Patients With Esophageal Cancer. Oncol Lett (2020) 19:487–97. 10.3892/ol.2019.11122 PMC6924116 31897162

[26] Suzuki R Lin SH Wei X Allen PK Welsh JW Byers LA. Prognostic Significance of Pretreatment Total Lymphocyte Count and Neutrophil-to-Lymphocyte Ratio in Extensive-Stage Small-Cell Lung Cancer. Radiother Oncol (2018) 126:499–505. doi: 10.1016/j.radonc.2017.12.030 29398150 PMC5856620

[27] Ho YC Lai YC Lin HY Ko MH Wang SH Yang SJ. Low Cardiac Dose and Neutrophil-to-Lymphocyte Ratio Predict Overall Survival in Inoperable Esophageal Squamous Cell Cancer Patients After Chemoradiotherapy. Sci Rep (2021) 11:6644. doi: 10.1038/s41598-021-86019-2 33758232 PMC7988072

[28] Dolladille C Ederhy S Sassier M Cautela J Thuny F Cohen AA. Immune Checkpoint Inhibitor Rechallenge After Immune-Related Adverse Events in Patients With Cancer. JAMA Oncol (2020) 6:865–71. doi: 10.1001/jamaoncol.2020.0726 PMC7163782 32297899

